# A new gamboge derivative Compound 2 inhibits cancer stem-like cells *via* suppressing EGFR tyrosine phosphorylation in head and neck squamous cell carcinoma

**DOI:** 10.1111/jcmm.12129

**Published:** 2013-09-23

**Authors:** Rongxin Deng, Xu Wang, Yang Liu, Ming Yan, Sayaka Hanada, Qin Xu, Jianjun Zhang, Zeguang Han, Wantao Chen, Ping Zhang

**Affiliations:** a Shanghai Key Laboratory of Stomatology, Shanghai Research Institute of StomatologyShanghai, China; b Department of Oral and Maxillofacial-Head & Neck Oncology Ninth People's Hospital, Shanghai Jiao Tong University School of MedicineShanghai, China; c School of Nursing, George Washington UniversityWashington, DC, USA

**Keywords:** cancer stem cell, EGFR, Compound 2, head and neck, squamous cell carcinoma, target therapy

## Abstract

Cancer stem-like cells represent a population of tumour-initiating cells that lead to the relapse and metastasis of cancer. Conventional anti-cancer therapeutic drugs are usually ineffective in eliminating the cancer stem-like cells. Therefore, new drugs or therapeutic methods effectively targeting cancer stem-like cells are in urgent need to successfully cure cancer. Gamboge is a natural anti-cancer medicine whose pharmacological effects are different from those of conventional chemotherapeutical drugs and they can kill some kinds of cancer cells selectively. In this study, we identified a new gamboge derivative, Compound 2 (C2), which presents eminent suppression effects on cancer cells. Interestingly, when compared with cisplatin (CDDP), C2 effectively suppresses the growth of both cancer stem-like cells and non-cancer stem-like cells derived from head and neck squamous cell carcinoma (HNSCC), inhibiting the formation of tumour spheres and colony *in vitro*, resulting in the loss of expression of multiple cancer stem cell (CSC)-related molecules in HNSCC. Treating with C2 effectively inhibited the growth of HNSCC in BALB/C nude mice. Further investigation found that C2 notably inhibits the activation of epithelial growth factor receptor and the phosphorylation of its downstream protein kinase homo sapiens v-akt murine thymoma viral oncogene homolog (AKT) in HNSCC, resulting in down-regulation of multiple CSC-related molecules in HNSCC. Our study has demonstrated that C2 effectively inhibits the stem-like property of cancer stem-like cells in HNSCC and may be a hopeful targeting drug in cancer therapy.

## Introduction

Head and neck squamous cell carcinoma is the six most common cancer in the world 1, with ∼600,000 patients newly diagnosed each year. Despite advances in the diagnosis and treatment of cancer, overall survival rates have not improved over the past few decades. Mortality in patients with HNSCC remains high because of regional and distant metastases and emergence of local and regional recurrences 2. Platinum-based chemotherapy combined with radiation and/or surgery is the standard treatment for local regional recurrences or metastatic disease. Currently, only around 35% of patients show response to this type of therapy and the response is usually short in duration, lasting 6–9 months 2–3. In random clinical trials, combination therapy is associated with greater toxicity and with no obvious increment in overall survival 4. A large meta-analysis of individual patient data has also reported that concurrent chemotherapy is not always associated with an improvement in overall survival in some patients 5. It has been found that one of the major causes of failure in HNSCC treatment is the enrichment of CSCs that are resistant to many current cancer treatments, including chemotherapy and radiotherapy 6.

In HNSCC, like many epithelial tumours, there is a subpopulation of cells with cancer stem-like cell properties, purported CSCs. It has been found that the multiple stem cell-related molecules are highly expressed in the HNSCC CSCs, including CD44 7, CD133 8, CD49f 9, ABCG2 10, Oct4 and Nanog 11. These CSCs have increasing self-renewal and tumour formation abilities; they are more inclined to be locally invasive, metastasis and be resistant to multiple traditional chemotherapy drugs, which lead to clinical chemotherapy failure, tumour local recurrence and distant metastasis 12. It has been suggested that in cases where chemotherapy or radiation treatment fails to completely eradicate the disease, the residual cancer cells will be highly enriched for cells that persist in a CSC state 13. Thus, these considerations indicate that to be effective in the long-term, successful cancer therapies need to include anti-cancer agents that take effect on CSCs to prevent the re-growth of cancer cell populations.

As a traditional Chinese medicine, gamboge is an effective as an anti-inflammatory agent, detoxication, coagulant and a strong apoptotic inducer in many types of cancer cells. Gambogic acid (GA) is the major active compound of gamboge 14. Gambogic acid has significant anti-tumourigenic characteristics and induces apoptosis of different types of cancer 15–19. Recent studies show that GA has great potential as an anti-cancer compound compared with conventional chemotherapeutical drugs because GA has shown to be highly selective in targeting cancer cells 20. This attribute increased attention towards GA; however, clinical limitations exist because GA half-life is very short 21–22 and presented overt toxicity during a phase II clinical trial. Many efforts have been made to enhance GA anti-tumorigenic effects, extend drug half-life and lower toxicity. In this study, our laboratory has identified a new gamboge derivative called Compound2 (C2; MW 633), which has a significantly longer half-life of 6 hrs and is more effective on cancer cells (Fig. S1). Importantly, different from the traditional chemotherapeutical drug cisplatin (CDDP), C2 effectively inhibits both CSCs and non-CSCs derived from HNSCC.

## Materials and methods

### Cell lines, cell cultures and reagents

Human HNSCC cell line Cal27 was obtained from American Type Culture Collection (Manassas, VA, USA); HN13, HN6, HN4, SCC25 were gifts from University of Maryland School of Dentistry; KB (ATCC CCL-17) and KB/Vincristine (VCR) were obtained from the Cell Bank of the Chinese Academy of Sciences Type Culture Collection Committee (Shanghai, China). All cell lines were cultured in DMEM (Gibco, Grand Island, NY, USA) containing 10% foetal bovine serum (FBS, Gibco), 1% glutamine and 1% penicillin-streptomycin at 37°C in a humidified atmosphere with 5% CO_2_. Compound 2 was synthesized in WuXi AppTec Co. Ltd (Shanghai, China), and its purity was 99.8% as testing with thing layer chromatography/high-performance liquid chromatography. Stock solution of C2 was prepared as 50 mmol/l in dimethyl sulfoxide (DMSO) and stored at −20°C.

### MTT assay

Cells were seeded in 96-wells plates at a density of 3 × 10^3^/well. After 24 h, cells were then treated with serial dilutions of C2 and GA, including a negative control with 2‰ DMSO. After an incubation period of 72 hrs, cell growth was determined with (3-(4,5-dimethylthiazol-2-yl)-2,5-diphenyltetrazolium bromide) (MTT) assay. The optical density value was measured at 490 and 630 nm as reference with an ELISA plate reader. The 50% inhibitory concentration (IC_50_) of C2 and GA was calculated with GraphPad5 software (Version 5.01, GraphPad Software, Inc., La Jolla, CA, USA).

### Western blot

Total proteins were collected in lysis buffer (1% Nonidet P-40, 5% sodium deoxycholate, 1 mM phenylmethanesulfonyl fluoride, 100 mM sodium orthovanadate) with protease inhibitor cocktail (Sigma-Aldrich, St. Louis, MO, USA) and stored at −20°C. Protein concentrations were determined by using the bovine serum albumin Protein Assay Kit (Pierce, Rockford, IL, USA). Twenty microgram of total proteins was separated on 8% SDS-PAGE and then transferred onto polyvinylidene difluoride membranes (Amersham Pharmacia Biotech, Piscataway, NJ, USA). After blocking with 5% non-fat milk, the membranes were then incubated with primary antibodies at 4°C overnight, which included: β-actin (1:10000 dilution, Sigma-Aldrich); CD133 (1:1000 dilution, PROTEINTECH, Chicago, IL, USA); CD49f (1:1000 dilution, Abcam, Cambridge, UK); CD44 (1:1000 dilution, EPITOMICS, Burlingame, CA, USA); Caspase3 (1:1000 dilution, Cell signaling, Danvers, MA, USA); PARP (1:1000 dilution, Cell signaling); Bcl-2 (1:1000 dilution, Cell signaling), phospho-AKT(Thr308) (1:1000 dilution, Cell Signaling), AKT (1:1000 dilution, Cell Signaling), Phospho-Erk1/2 (Thr202/Tyr204) (1:1000 dilution, Cell Signaling), Erk1/2 (1:1000 dilution, Cell Signaling), phospho-EGFR (Try1068) (1:1000 dilution, Cell Signaling) and EGFR (1:1000 dilution, Cell Signaling). Secondary antibodies goat anti-mouse or goat anti-rabbit (1:15000 dilutions, LI-COR Biotechnology, Lincoln, NE, USA) were used for 1 hr at room temperature. Finally, the membranes were scanned and analysed with the Odyssey Infrared Imaging System (LI-COR Biosciences).

### Real-time PCR

Cells were harvested at 24 or 48 hrs after treatment. Total RNA was extracted by using TRIzol reagent (Invitrogen, Carlsbad, CA, USA) and converted to cDNA by using TaKaRa cDNA synthesis kit (TaKaRa, Dalian, China). Real-time PCR reactions were performed with the SYBR Premix Ex Taq™ reagents kit (Takara). The primer sequences included CD44: sense 5′-TAACCGTGATGGCACCCGCT-3′ and anti-sense 5′-TTGAAGACGTACTGGTAGCAGGGA-3′; CD133: sense 5′-GCATGCAAAAGCCATCATAG-3′ anti-sense 5′-GGGAATGCCTACATCTGGAA-3′; Oct-4: sense 5′-CGCACCACTGGCATTG TCAT-3′ anti-sense 5′-TTCTCCTTGATGTCACGCAC-3′; ABCG2: sense 5′-CTGAGATCCTGAGCCTTTGG-3′ anti-sense 5′-TGCCCATCACAAC ATCATCT-3′; Nanog: sense 5′-AATACCTCAGCCTCCAGCAGATG-3′ anti-sense 5′-CTGCGTCACACCATTGCTATTCT-3′; β-actin: sense 5′-CCTGGCACCCAGCACAAT-3′ anti-sense 5′-GGGCCGGACTCGTCATACT-3′. Results of real-time PCR were represented as ΔΔCt values and normalized with the Ct of β-actin. A *P*-value under 0.05 was defined as significance.

### Cell apoptosis analysis

All cells were harvested at 24 hrs after drug treatment or DMSO treatment, stained with Annexin V- PI Apoptosis Detection Kit (BD Biosciences, San Jose, CA, USA) and then analysed on FACSCalibur (BD Biosciences), according to the manufacturer^'^s protocols.

### Rhodamine 123 cell staining

Cells were seeded on slides in 24 well plates. After being treated with C2 for 24 hrs, cells were incubated at 37°C with 200 μl/well Rh123 (1:1000 dilutions) for 2 hrs, fixed with 4% para-formaldehyde for 10 min., stained with polyvinylidene difluoride for 5 min., and then mounted on microscopic glasses with 70% glycerol. The slides were observed with a Leica TCS SP2 confocal spectral microscope (Buffalo Grove, IL, USA) and three different random fields were observed at 200× magnification.

### Colony formation and anchorage-independent growth in soft agar

Cal27 were plated at 2000 cells in 6 cm plates and treated with or without C2. Ten days later, the plates were stained with Coomassie Brilliant Blue dye for 30 min. and washed with PBS. For the soft agar assay, the bottom agar consisted of 1.2% low melting agarose with DMEM and 10% FBS. The top agar was made from 2000 cells mixed with 1.5 ml of 0.7% agar with DMEM and 10% FBS. 0.5 ml DMEM was added on top of the solidified agar layers, where the colonies were allowed to grow for 2 weeks.

### Sphere formation assay

Tumour sphere assay was performed as previously described. Cells were seeded in a low-adhesion 10-cm plate at a density of 5000 cells. Spheres were grown in DMEM medium (Gibco) supplemented with 20 ng/ml human EGF and 15 ng/ml human bFGF. EGF and bFGF were replenished every 3 days. Spheres were counted and photographed after 14 days.

### Flow cytometry analysis

Cells were resuspended in PBS at the concentration of 2 × 10^7^/ml. The cell suspensions were stained with anti-CD133 (1:1000 dilution, PROTEINTECH), anti-CD49f (1:1000 dilution, Abcam, Cambridge, UK), or isotype control on ice for 30 min. in the dark, centrifuged at 300 × *g* for 5 min., added the appropriate FITC-labelled second antibody at 1:100 dilution and incubated for another 15 min. on ice in the dark. Analysis was performed on FACSCalibur (BD Biosciences, San Jose, CA, USA) flow cytometry.

### Isolation of CD133 positive cells

1 × 10^8^ cells were collected and resuspended in 300 μl binding buffer with 100 μl Fc receptor blocking reagent and 100 μl CD133 microbeads at 4°C for 30 min. (Miltenyi Biotech, Aubum, CA, USA). The samples were then loaded on MS columns (Miltenyi Biotech) and CD133^−^ and CD133^+^ cells were separated with MACS Cell Separation (Miltenyi Biotech).

### Tumour-transplanted model and *in vivo* treatment

*In vivo* experiments were performed in accordance with the institutional guidelines for the use of laboratory animals. Four-week-old BALB/C nude female mice were supplied by the Shanghai Experimental Animal Center, Chinese Academy of Sciences, Shanghai, China. Cal27 cells in the exponential phase were trypsinized, washed with DMEM and suspended in PBS to obtain a concentration of 1 × 10^7^. Subsequently, 200 μl of suspended cells was subcutaneously inoculated into flanks of each nude mouse bilaterally. The mice were randomly divided into five groups, consisting of three mice in each group. The first group served as negative controls, receiving no treatment. The second group, C2 50 mg/kg group, received oral administration of 50 mg/kg C2 per day. The third group, C2 100 mg/kg group, received oral administration of 100 mg/kg C2 per day. The fourth group, CDDP group, was treated with intraperitoneal injections of 5 mg/kg CDDP every other day. The fifth group, Lap group, received oral administration of 100 mg/kg lapatinib every other day. Every other day, mice were weighed and the volumes of the tumours were measured. Treatment was initiated when the majority of the tumours had reached a volume of 20 mm^3^ on the 7th day. Thirty days after drug therapy administration, mice were killed and the tumours were excised, fixed with 10% formalin and paraffin-embedded.

### Immunohistochemical staining

The paraffin blocks of xenograft tumours were cut into 5 μm sections for standard immunohistochemical staining (IHC). After heat-induced antigen retrieval in citric acid buffer (pH 7.0) for 18 min. and blocked in H_2_O_2_ solution for 15 min., slides were then incubated with rabbit anti- Ki-67, rabbit anti- CD49f, rabbit anti- CD133, rabbit anti-phospho-EGFR (Tyr1068) at a dilution of 1:100 at 4° overnight. Bound antibody was detected by a Super Sensitive IHC Detection System (BioGenex, Fremont, CA, USA). The sections were visualized by using diaminobenzidine tetrahydrochloride (Sigma-Aldrich) solution and counter stained with Harris haematoxylin. Staining was then scored by an experienced pathologist blinded to the treatment groups.

## Results

### Compound 2 effectively inhibited the growth of cancer cells and induced apoptosis, while exerted only marginal effect on normal cells

**Figure 1:**
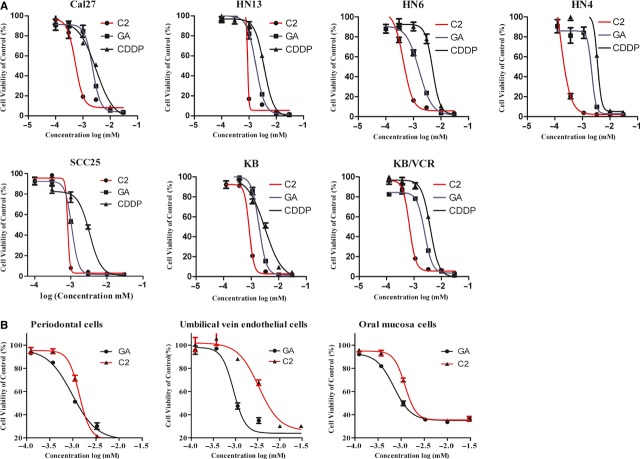
Inhibitory effects of C2 on head and neck squamous cell carcinoma (HNSCC) cells. (A) The 50% inhibitory concentration (IC_50_) of C2 and CDDP on different HNSCCs. (B) The inhibitory effects of C2 on normal cells (from left to right: periodontal cells, umbilical vein endothelial cells and oral mucosa cells).

To study the effect of C2 on cancer cells, we first examined the viability of seven cancer cells treated with gradient dosages of C2 for 72 hrs with MTT assay. As shown in Figure 1A, the inhibitory efficacy of C2 on cancer cells was more successful compared with the traditional chemotherapeutic drug CDDP. The IC_50_ of C2 on different cancer cells was from 0.144 μM (HN4) to 0.885 μM (HN13), with median value 0.59 μM, while the IC_50_ of CDDP was from 3.065 μM (Cal 27) to 4.881 μM (KB/VCR), with median value 3.839 μM (Table S1). Some cancer cells showed less sensitive to C2, while others were more sensitive, indicating selective effects of C2 on different cells, while the IC_50_ of CDDP was more consistent across all cancer cell lines. We also examined the toxicity of C2 on various primary cultured normal cells, including periodontal cells, umbilical vein endothelial cells, and oral mucosa cells. As Figure 1B shows, the IC_50_ of C2 on normal cells was from 1.212 to 3.345 μM, with median 1.98 μM, which was about three times higher than the IC_50_ of C2 on cancer cells.

**Figure 2:**
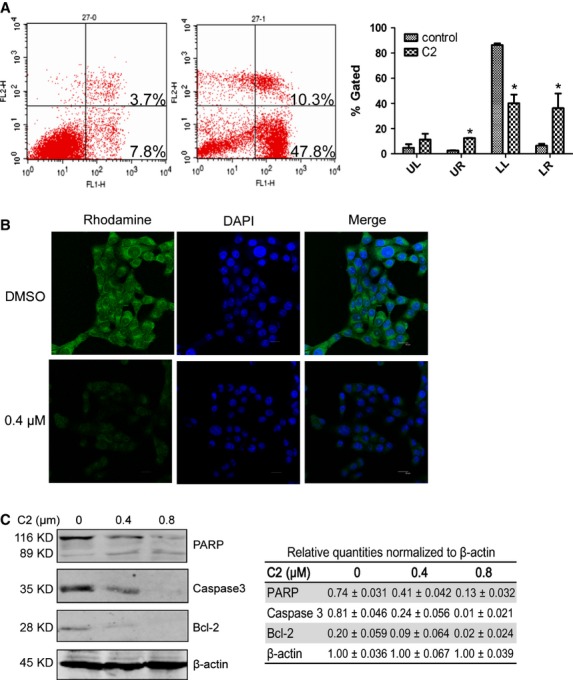
C2 induced apoptosis of cancer cells. (A) Annexin-V/PI double staining shows that treatment with 0.8 μM C2 for 24 hrs, induced apoptosis in 58.1% of Cal27 cells compared with only 11.5% in DMSO-treated group. (B) Treatment with 0.4 μM C2 significantly reduced the mitochondrial membrane potential of Cal27 cells. (C) The expression of full length PARP, full length Caspase3 and Bcl-2 were remarkably decreased in a drug concentration-dependent manner. Error bars represent SD. * indicates *P* < 0.05.

Annexin-V/PI double staining showed that treatment with C2 for 24 hrs induced eminent apoptosis of HNSCC cells, as Figure 2A shows that the apoptotic rate was 58.1% in Cal27 treated with 0.8 μM C2, compared with 11.5% in cells treated with DMSO. Rh123 cell staining demonstrated that 24-hr treatment with C2 significantly reduced the mitochondrial membrane potential. Figure 2B shows that the mitochondrial membrane potential was significantly reduced in Cal27 treated with 0.4 μM C2. We further examined the expression of full length Caspase 3, PARP and Bcl-2 in HNSCC cells treated with C2. As Figure 2C shows, full length of PARP and full length of Caspase3 were decreased in a dose-dependent manner. Bcl-2 was also decreased in C2-treated Cal27 cells in a dose-dependent manner. The expression levels of apoptosis-related proteins tested by western blot supported that C2 induced apoptosis of HNSCC cells.

### Compound 2 inhibited colony formation ability and anchorage-independent growth of cancer cells

**Figure 3:**
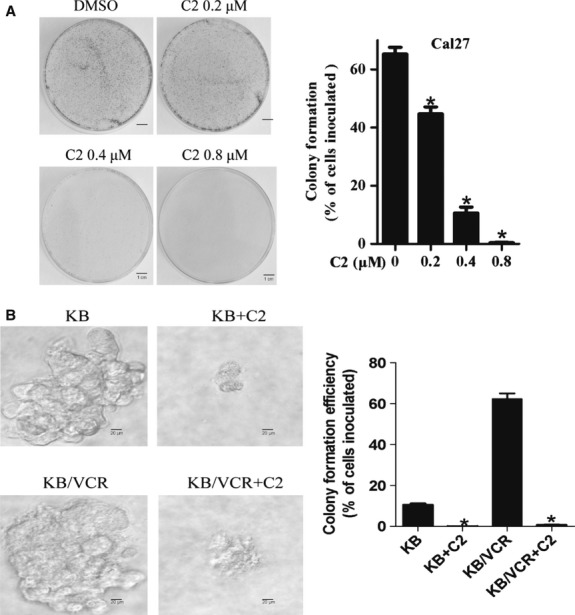
C2 significantly inhibited colony formation ability and anchorage-independent growth of cancer cells. (A) C2 inhibited the colony formation ability of Cal27 cells. (B) C2 consistently inhibited the anchorage-independent growth in soft agar of both drug-sensitive KB cells and drug-resistant KB/VCR cells, which indicated that C2 might be able to target cancer stem cells. Error bars represent SD. * indicates *P* < 0.05.

As the efficacy of colony formation and anchorage-independent growth primarily reflected the self-renewal ability of cancer cells *in vitro*, we tested the influence of C2 on these abilities in several HNSCC cell lines, including Cal27, KB, and KB/VCR cells. We found that C2 significantly reduced the colony formation ability of these HNSCC cells in a dose-dependent manner (Fig. 3A). The soft agar assay confirmed the inhibitory efficacy of C2 on the self-renewal ability of cancer cells (Fig. 3B). Interestingly, as Figure 3B shows, C2 exerted similar inhibitory effect on the anchorage-independent growth of both drug-resistant KB/VCR and drug-sensitive KB cells.

### Compound 2 effectively exerted inhibitory effects on HNSCC CSCs

**Figure 4:**
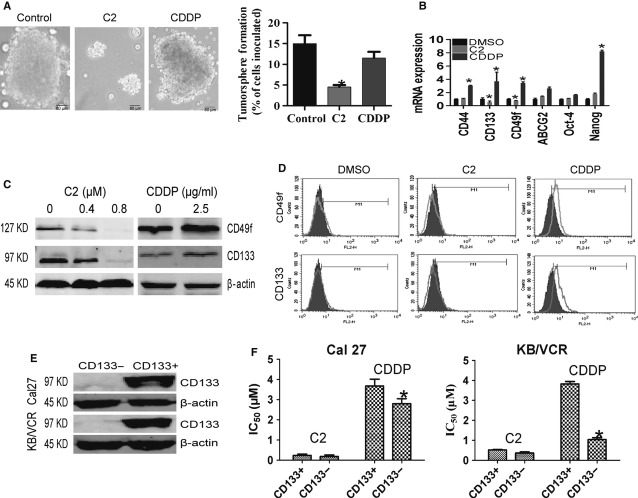
C2 effectively exerted inhibitory effects on head and neck squamous cell carcinoma cancer stem cells. (A) C2 remarkably suppressed tumour sphere formation of Cal27 cells in serum-free medium, while CDDP failed to do so. (B) CDDP treatment led to increases in multiple stem cell-related molecules, including CD44, CD133, CD49f and Nanog, while no such increases were observed in the C2-treated group. On the contrary, the mRNA levels of CD133 and CD49f were remarkably reduced in C2-treated group. (C) Western blot confirmed that C2 inhibited the expression of CD49f and CD133 in a dose-dependent manner in Cal27 cells, while CDDP somehow increased their expressions. (D) Flow cytometry analysis revealed that CD49f^+^ and CD133^+^ subpopulations were suppressed by C2 treatment, but were enriched by nearly 50% by CDDP treatment. (E) CD133^+^ and CD133^−^ Cal27 and KB/VCR cells were isolated. (F) The sensitivity to C2 treatment was tested. There was no significant difference between the 50% inhibitory concentration (IC_50_) of C2 in CD133^+^ populations and CD133^−^ populations; however, CD133^−^ cells were much more sensitive to CDDP compared with CD133^+^ cells. Error bars represent SD. * indicates *P* < 0.05.

As chemo-resistance is a typical feature of HNSCC CSCs and our former results showed that C2 treatment resulted in a reduction in colony-forming efficiency of both drug-resistant and -sensitive HNSCC cancer cells, we further investigated the inhibitory efficacy of C2 on HNSCC CSCs. Firstly, we examined the influence of C2 on the sphere formation ability of Cal27 cells in serum-free medium, as surrogate readout of stem-cell abundance. Consistently, we found that C2 significantly inhibited the sphere formation ability of Cal27 cells, while traditional chemotherapeutic drug CDDP had no obvious inhibition effect (Fig. 4A). Then, we investigated whether treatment with C2 influenced the expression of stem cell-related molecules in cancer cells. As Figure 4B shows, after 24-hr treatment with CDDP, most tested stem cell-related molecules, including CD44, CD133, CD49f and Nanog were significantly up-regulated in CDDP-treated group, but no such increases were observed in C2-treated group. On the contrary, the mRNA levels of CD133 and CD49f were remarkably reduced in C2-treated group. Western blot confirmed that C2 inhibited the expression of CD49f and CD133 in a dose-dependent manner in Cal27 cells, while CDDP somehow increased the expression of CD49f and CD133 in Cal27 cells (Fig. 4C). Flow cytometry analysis further revealed that 0.4-μM C2 treatment for 48 hrs suppressed these CD49f^+^ and CD133^+^ subpopulations in Cal27 cells, while 2.5 μg/ml CDDP treatments for 48 hrs significantly augmented these subpopulations from 7.50% to 46.15% (Fig. 4D). As CD133 is a commonly used CSC marker in various types of cancer, we then isolated CD133^+^ and CD133^−^ subpopulations from Cal27 and KB/VCR cells (Fig. 4E) and compared their sensitivity with C2 treatment. As Figure 4F and Table S2 show, there are no significant differences between the IC_50_ of C2 in CD133^+^ cells and the IC_50_ of C2 in CD133^−^ cells. However, CD133^−^ cells were increasingly more sensitive to CDDP compared with CD133^+^ cells derived from both Cal27 and KB/VCR. Therefore, compared with conventional chemotherapeutic CDDP regimen, C2 exhibited a significantly increased effective inhibitory effect on HNSCC CSCs.

### Compound 2 exerted inhibitory effect on HNSCC *in vitro via* repressing the activation of EGFR pathway

**Figure 5:**
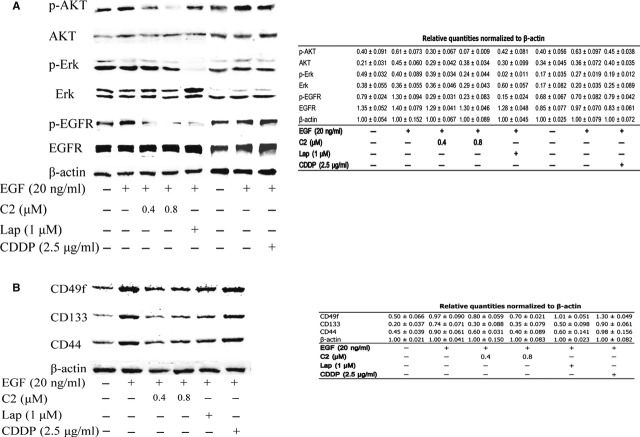
C2 exerted inhibitory effect on head and neck squamous cell carcinoma (HNSCC) *in vitro via* repressing the activation of EGFR pathway. (A) As EGFR kinase can promote acquisition of cancer stem-cell (CSC) properties in HNSCC and studies have also found overexpression of EGFR resulted in an increased expression of multiple stem-cell marker, so we compared the influence of C2 and CDDP on the activation of EGFR signalling pathway. Small molecular inhibitor of EGFR lapatinib was used as a positive control. Western blot analysis revealed that after 24-hr treatment with C2, the stimulation effect of EGF on the EGFR phosphorylation and the activation of EGFR downstream AKT were significantly inhibited by C2 in a dose-dependent manner, while CDDP failed to do so, and lapatinib also significantly inhibited the EGF phosphorylation and the activation of AKT induced by EGF. (B) Western blot analysis showed that EGF treatment significantly induced the up-regulation of multiple stem cell-related molecules in cancer cells; while in C2 treatment and lapatinib treatment groups, the EGF induced up-regulation of stem cell-related molecules were all blocked; yet, no such inhibition has been observed in CDDP treatment group. The data indicate that the efficacy of C2 on CSCs is largely through its inhibitory effect on EGF/EGFR signal pathway, resulting in loss of expression of multiple CSC markers in HNSCC. The relative quantities of each blot were normalized to the value of β-actin and listed in the Tables.

As increasing amounts of data demonstrate that the activation of EGFR pathway plays an indispensible role in the maintenance and proliferation of stem cells by regulating the expression of multiple stem cell-related molecules 23, we investigated the effect of C2 on the activation of EGFR pathway. Binding of EGFR to its ligands EGF leads to autophosphorylation of tyrosine residues on the EGFR and subsequent activation of signal transduction pathways that involved in regulating cellular proliferation, differentiation and survival. Phosphorylation of Akt and Erk are two most widely studied transduction molecules triggered by activation of EGFR; while in our study, as Figure 5A shows, after 24-hr treatment with C2, the stimulation effect of EGF on EGFR phosphorylation and the activation of EGFR downstream Akt were significantly inhibited by C2 in a dose-dependent manner, while after 48-hr treatment with C2, the EGF induced up-regulation of stem cell-related molecules, including CD49f, CD133 and CD44 were significantly blocked (Fig. 5C). Similar results were observed in lapatinib-treated cells, which is a small molecule inhibitor of EGFR tyrosine-kinases. Consistently, CDDP treatment failed to inhibit the stimulation of EGF on EGFR signalling and resulted in the up-regulation of CD49f, CD133 and CD44.

### Compound 2 exerted a significant inhibitory effect on HNSCC xenograft tumours and suppressed the EGFR phosphorylation *in vivo*

**Figure 6:**
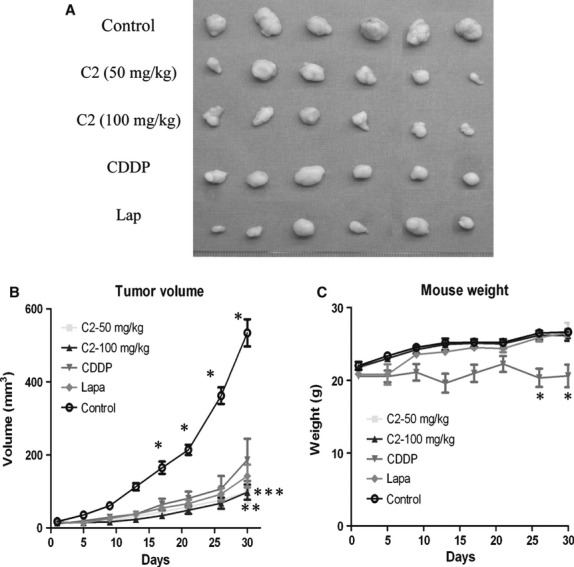
Inhibitory effects of C2 on Cal27-derived xenografted tumours. (A) Oral administration of C2 effectively inhibited the tumour growth *in vivo*. The inhibitory effect of C2 100 mg/kg treatment was the strongest positive compared with other treatment groups. (B) The tumour growth curve of each treatment group. * indicates significant difference between control group and treated group. ** indicates significant difference between CDDP group and C2 50 mg/kg group. *** indicates significant difference between CDDP group and C2 100 mg/kg group. (C) The weight changes of each different treatment group. The CDDP treatment group significantly lost overall body weight, while there were no remarkable body weight changes in the other treatment groups. Error bars represent SD. * indicates *P* < 0.05.

**Figure 7:**
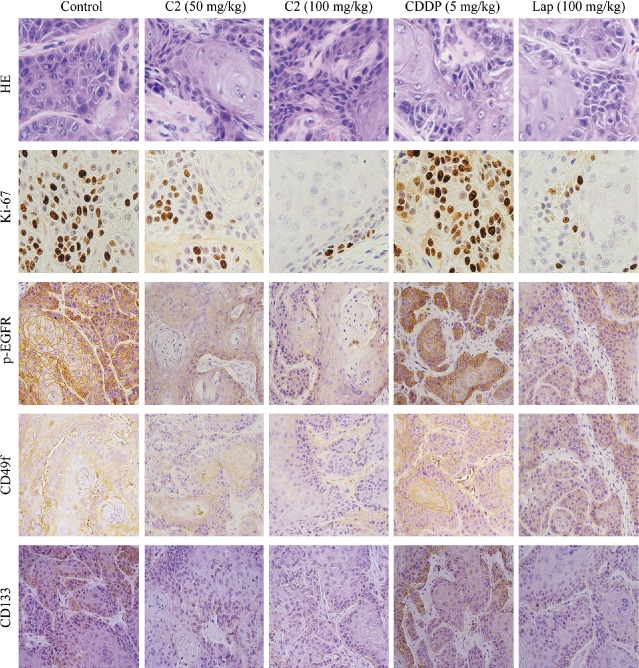
Haematoxylin and eosin staining and immunohistochemical assay of xenografted tumours. C2 effectively inhibited the expression of Ki-67, phosphor-EGF, CD49f and CD133 in xenograft tumours in a dose-dependent manner, while CDDP failed to exert such inhibitory effects.

To evaluate the *in vivo* effect of C2 on HNSCC therapy, we used Cal27 cells to establish transplanted tumours in nude mice. When the xenograft tumours averaged 20 mm^3^ in size, the mice were randomly divided into five different treatment groups, with three mice assigned to each group. In the blank control group, the tumour grew very quickly without any drug treatment and reached more than 500 mm^3^ at the 30th day after inoculation. In all four treatment groups with CDDP, C2, or lapatinib, tumour growths were significantly inhibited, as shown in Figure 6A and B. The inhibitory effect on the implanted tumours in the C2 100 mg/kg group had the best outcome amongst all treatment groups, while the tumours generated in both the C2 50 mg/kg group and C2 100 mg/kg group were significantly smaller in size than tumours generated in CDDP group (*P* < 0.05, Fig. 6B). As shown in Figure 6C, during the treatment the mice in C2 50 mg/kg, C2 100 mg/kg, lapatinib and blank control groups had steadily gained weight, while mice in CDDP group did not gain weight at all or slightly lost body weight. Immunohistochemical staining of the excised transplanted tumours (Fig. 7) further validated that similarity to the lapatinib treatment group; C2 effectively inhibited the expression of Ki-67, phosphor-EGFR, CD49f and CD133 in cancer cells in a dose-dependent manner, while CDDP failed to exert such inhibitory effects.

## Discussion

Patients with non-treatable advanced recurrent or metastatic HNSCC have a median survival of 6 months 4. Combination platinum-based chemotherapy has been the standard of care, but large meta-analysis of individual patient data has reported that concurrent chemotherapy is not associated with an improvement in overall survival in patients with HNSCC 5. As shown here and elsewhere 13–24, treatment with conventional chemotherapeutics actually imposes a positive selection for CSC survival and expansion. We found that after CDDP treatment, HNSCC have an increased proportion of cells with CD133^+^ and CD49f^+^ and increased expression of CD44, CD133, CD49f, and Nanog. This suggests that when chemotherapy fails to completely eradicate the disease, the residual cancer cells will be enriched for cells that carry a CSC feature. These observations indicate that cancer therapies should include agents that can effectively target CSCs.

In this study, we identified gamboge derivative C2, which presents a hopeful anti-cancer treatment candidate that can be used to target CSCs. As a promising anti-tumourigenic candidate, C2 has a prolonged half-life and with greatly enhanced tumour inhibitory effects, while having mild toxicity to normal cells and can be orally administrated. *In vivo*, the anti-tumourigenic effects of C2 were eminent and the inhibition efficacy of oral administration of 50 mg/kg C2 or 100 mg/kg C2 alone was vastly improved compared with that of the peritoneal administration of 5 mg/kg CDDP. At the same time, there was no obvious adverse effect of C2 on the weight gain of those treatment groups, unlike the CDDP treatment group experienced. It is of note that the inhibitory effect of C2 on drug-resistant KB/VCR cells was very close to on the drug-sensitive KB cells, while the normal cells were insensitive to C2 treatment, which made C2 an attractive cancer selective drug. Our further investigation revealed that different from CDDP treatment, the C2 treatment effectively inhibited the expression of multiple stem cell-related molecules, suppressed colony formation efficiency and decreased the anchorage-independent growth in soft agar and sphere-forming ability. CD133 is a widely used CSC marker 25–26. Compared with CD133^−^ HNSCC cells, CD133^+^ CSC-like cells are more resistant to CDDP treatment, while we found that C2 effectively inhibited the growth of both CD133^+^ and CD133^−^ HNSCC cells. These observations indicate that C2 is different from conventional chemotherapeutic drugs and treatment with C2 will not lead to selectively enrich cancer cells with CSC features. The *in vivo* treatment with C2 or CDDP on transplanted animal models further confirmed these observations as only C2 treatment effectively suppressed the expression of multiple CSC-related molecules both *in vivo* and *in vitro*.

The transmembrane protein, EGFR, has an intrinsic tyrosine kinase activity and regulates cell growth in response to binding of ligand like EGF 27. This pathway is a potent regulator of tumour cell growth, invasion, angiogenesis and metastasis. It is overexpressed in almost all HNSCC tumours and its overexpression is associated with poor prognosis 28. A recent study indicated that EGFR kinase promoted acquisition of CSC properties in HNSCC. Overexpression of EGFR resulted in an increased expression of stem-cell marker, CD44, Oct-4, and Nanog 29, so EGFR inhibition might be an effective method to target the CSC populations in HNSCC. It has been reported that small molecule inhibitors of EGFR, such as lapatinib, erlotinib, gefitinib and cetuximab, can prevent the growth and progression of HNSCC and decrease the expression of stem cell-related molecule. Cetuximab has recently been approved for HNSCC treatment 30. Here, we found that treatment with C2 for 24 hrs can effectively block the stimulation effects of EGF on HNSCC cells in a dose-dependent manner. In C2-treated cells, EGF fails to initiate the phosphorylation of EGFR, resulting in significantly decreased expression of phosphory-Akt and slightly decreased expression of phosphory-Erk1/2. Also, 48 hrs after C2 treatment, similar to the lapatinib treatment, multiple stem cell-related molecules, such as CD133, CD49f and CD44 were down-regulated; yet, no such inhibition has been observed in CDDP treatment group. This observation demonstrates that the efficacy of C2 on CSCs is largely through its inhibitory effect on EGF/EGFR signal pathway and C2 not only effectively inhibited the *in vivo* growth of transplanted HNSCC but also blocked the activation of EGFR signal and resulted in loss of expression of multiple CSC markers in HNSCC. C2 may be a useful adjuvant to current chemotherapeutic drug.

## Conclusion

We identified a new Gamboge derivative called C2, which presented stronger and more effective anti-tumourigenic activity against HNSCC. Notably, C2 can effectively target both CSCs and non-CSCs within HNSCC, which makes it different from conventional chemotherapeutic drugs, in that it prevents the selective enrichment of CSCs after chemotherapeutic drug treatment. The inhibitory effect of C2 on CSCs largely occurs through blocking the activation of EGF/EGFR signal pathway, which leads to down-regulation of multiple stem cell-related molecules. These features make C2 a promising candidate for use in long-term and recurrent cancer drug therapies.

## Supporting Information

**Table S1** 50% inhibiting concentration (IC_50_) of C2, CDDP and GA on cancer cells and normal cells. **Table S2** 50% inhibiting concentration (IC_50_) of C2 and CDDP on CD133^+^ and CD133^-^ HNSCC cells. **Fig. S1** The half-life of C2 and GA was detected by metabolic stability assay combined with LS/MS/MS assay. GA or C2 were incubated with rat liver microsomes for indicated time and the concentration was determined with LC/MS/MS. The half-life of GA and C2 was 3.1 and 6.3 hrs, respectively.

## References

[b1] Nemunaitis J, O'Brien J (2002). Head and neck cancer: gene therapy approaches. Part II: genes delivered. Expert Opin Biol Ther.

[b2] Cripps C, Winquist E, Devries MC (2010). Epidermal growth factor receptor targeted therapy in stages III and IV head and neck cancer. Curr Oncol.

[b3] Gupta PB, Onder TT, Jiang G (2009). Identification of selective inhibitors of cancer stem cells by high-throughput screening. Cell.

[b4] Bourhis J (2005). New approaches to enhance chemotherapy in SCCHN. Ann Oncol.

[b5] Pignon JP, Maitre le A, Bourhis J (2007). Meta-analyses of chemotherapy in head and neck cancer (MACH-NC): an update. Int J Radiat Oncol Biol Phys.

[b6] Diehn M, Cho RW, Lobo NA (2009). Association of reactive oxygen species levels and radioresistance in cancer stem cells. Nature.

[b7] Prince ME, Sivanandan R, Kaczorowski A (2007). Identification of a subpopulation of cells with cancer stem cell properties in head and neck squamous cell carcinoma. Proc Natl Acad Sci USA.

[b8] Zhang Z, Filho MS, Nor JE (2012). The biology of head and neck cancer stem cells. Oral Oncol.

[b9] Bragado P, Estrada Y, Sosa MS (2012). Analysis of marker-defined HNSCC subpopulations reveals a dynamic regulation of tumor initiating properties. PLoS ONE.

[b10] Zhang P, Zhang Y, Mao L (2009). Side population in oral squamous cell carcinoma possesses tumor stem cell phenotypes. Cancer Lett.

[b11] Chiou SH, Yu CC, Huang CY (2008). Positive correlations of Oct-4 and Nanog in oral cancer stem-like cells and high-grade oral squamous cell carcinoma. Clin Cancer Res.

[b12] Tsai LL, Yu CC, Chang YC (2011). Markedly increased Oct4 and Nanog expression correlates with cisplatin resistance in oral squamous cell carcinoma. J Oral Pathol Med.

[b13] Li X, Lewis MT, Huang J (2008). Intrinsic resistance of tumorigenic breast cancer cells to chemotherapy. J Natl Cancer Inst.

[b14] Asano J, Chiba K, Tada M (1996). Cytotoxic xanthones from Garcinia hanburyi. Phytochemistry.

[b15] Guo QL, You QD, Wu ZQ (2004). General gambogic acids inhibited growth of human hepatoma SMMC-7721 cells *in vitro* and in nude mice. Acta Pharmacol Sin.

[b16] Liu W, Guo QL, You QD (2005). Anticancer effect and apoptosis induction of gambogic acid in human gastric cancer line BGC-823. World J Gastroenterol.

[b17] Bai J, Sui J, Demirjian A (2005). Predominant Bcl-XL knockdown disables antiapoptotic mechanisms: tumor necrosis factor-related apoptosis-inducing ligand-based triple chemotherapy overcomes chemoresistance in pancreatic cancer cells *in vitro*. Cancer Res.

[b18] Regan PL, Jacobs J, Wang G (2011). Hsp90 inhibition increases p53 expression and destabilizes MYCN and MYC in neuroblastoma. Int J Oncol.

[b19] Kim RH, Kim R, Chen W (2008). Association of hsp90 to the hTERT promoter is necessary for hTERT expression in human oral cancer cells. Carcinogenesis.

[b20] Davenport J, Manjarrez JR, Peterson L (2011). Gambogic acid, a natural product inhibitor of Hsp90. J Nat Prod.

[b21] Liu YT, Hao K, Liu XQ (2006). Metabolism and metabolic inhibition of gambogic acid in rat liver microsomes. Acta Pharmacol Sin.

[b22] Yang J, Ding L, Hu L (2011). Metabolism of gambogic acid in rats: a rare intestinal metabolic pathway responsible for its final disposition. Drug Metab Dispos.

[b23] Jiang H, Grenley MO, Bravo MJ (2011). EGFR/Ras/MAPK signaling mediates adult midgut epithelial homeostasis and regeneration in Drosophila. Cell Stem Cell.

[b24] Fillmore CM, Kuperwasser C (2008). Human breast cancer cell lines contain stem-like cells that self-renew, give rise to phenotypically diverse progeny and survive chemotherapy. Breast Cancer Res.

[b25] Zhang Q, Shi S, Yen Y (2010). A subpopulation of CD133(+) cancer stem-like cells characterized in human oral squamous cell carcinoma confer resistance to chemotherapy. Cancer Lett.

[b26] Chen YS, Wu MJ, Huang CY (2011). CD133/Src axis mediates tumor initiating property and epithelial-mesenchymal transition of head and neck cancer. PLoS ONE.

[b27] Sheikh Ali MA, Gunduz M, Nagatsuka H (2008). Expression and mutation analysis of epidermal growth factor receptor in head and neck squamous cell carcinoma. Cancer Sci.

[b28] Rubin Grandis J, Melhem MF, Barnes EL (1996). Quantitative immunohistochemical analysis of transforming growth factor-alpha and epidermal growth factor receptor in patients with squamous cell carcinoma of the head and neck. Cancer.

[b29] Chen JS, Pardo FS, Wang-Rodriguez J (2006). EGFR regulates the side population in head and neck squamous cell carcinoma. Laryngoscope.

[b30] Leeman-Neill RJ, Seethala RR, Singh SV (2011). Inhibition of EGFR-STAT3 signaling with erlotinib prevents carcinogenesis in a chemically-induced mouse model of oral squamous cell carcinoma. Cancer Prev Res.

