# Supplementary material for: A new gamboge derivative Compound 2 inhibits cancer stem-like cells via suppressing EGFR tyrosine phosphorylation in head and neck squamous cell carcinoma
Source: J Cell Mol Med. 2013 Sep 23;17(11):1422–33. doi: 10.1111/jcmm.12129 (PMC4117555; doi:10.1111/jcmm.12129)
Supplement: Supplementary file 1 — Table S1 50% inhibiting concentration (IC50) of C2, CDDP and GA on cancer cells and normal cells. Table S2 50% inhibiting concentration (IC50) of C2 and CDDP on CD133+ and CD133- HNSCC cells. Fig. S1 The half-life of C2 and GA was detected by metabolic stability assay combined with LS/MS/MS assay. GA or C2 were incubated with rat liver microsomes for indicated time and the concentration was determined with LC/MS/MS. The half-life of GA and C2 was 3.1 and 6.3 hrs, respectively. [file jcmm0017-1422-sd1.docx]

**Supplementary Data**

**Table S1** 50% inhibiting concentration (IC_50_) of C2, CDDP, and GA on cancer cells and normal cells.

| \|  \| \| \| \| \| \|  \| IC_50_ ±SD(μM) \| \| \| \| --- \| --- \| --- \| --- \| --- \| --- \| --- \| --- \| --- \| --- \| \|  \| \| \| \| \| \|  \| C2 \| CDDP \| GA \| \| Cal27 \| \| \| \| \| \| \| 0.5285±0.04281 \| 3.065±0.0.04211 \| 2.257±0.05189 \| \| HN13 \| \| \| \| \| \| \| 0.8885±0.03178 \| 3.609±0.06112 \| 1.784±0.01452 \| \| HN6 \| \| 0.433±0.044 \| 4.965±0.01531 \| 1.617±0.03047 \| \| HN4 \| \| \| 0.144±0.075 \| 3.527±0.03524 \| 2.173±0.04724 \| \| SCC25 \| \| \| \| 0.82±0.013 \| 3.315±0.03126 \| 1.064±0.05179 \| \| KB \| \| \| \| \| 0.577±0.028 \| 3.165±0.04128 \| 1.951±0.01353 \| \| KB/VCR \| \| \| \| \| \| 0.679±0.058 \| 4.881±0.105 \| 2.462±0.02080 \| \| Periodontal cells \| \| \| \| \| \| \| 1.384±0.0178 \|  \| 0.968±0.02145 \| \| Umbilical vein endothelial cells \| \| \| \| \| \| \| 3.345±0.04215 \|  \| 0.8985±0.03581 \| \| Oral mucosa cells \| \| \| \| \| \| \| 1.212±0.01432 \|  \| 0.695±0.03512 \| |  |  | |
| --- | --- | --- | --- | --- | --- | --- | --- | --- | --- | --- | --- | --- | --- | --- | --- | --- | --- | --- | --- | --- | --- | --- | --- | --- | --- | --- | --- | --- | --- | --- | --- | --- | --- | --- | --- | --- | --- | --- | --- | --- | --- | --- | --- | --- | --- | --- | --- | --- | --- | --- | --- | --- | --- | --- | --- | --- | --- | --- | --- | --- | --- | --- | --- | --- | --- | --- | --- | --- | --- | --- | --- | --- | --- | --- | --- | --- | --- | --- | --- | --- | --- | --- | --- | --- | --- | --- | --- | --- | --- | --- | --- | --- | --- | --- | --- | --- | --- | --- | --- | --- | --- | --- | --- | --- | --- | --- | --- | --- |
|  |  |  |  |

| **Table S2** 50% inhibiting concentration (IC_50_) of C2 and CDDP on CD133^+^ and CD133^-^ HNSCC cells.   \|  \| **IC_50_ ±SD(μM)** \| \| \| --- \| --- \| --- \| \|  \| **C2** \| **CDDP** \| \| **Cal27** \|  \|  \| \| **CD133+** \| 0.237±0.064 \| 3.672±0.343 \| \| **CD133-** \| 0.187±0.072 \| 2.803±0.247 \| \| **KB/VCR** \|  \|  \| \| **CD133+** \| 0.517±0.025 \| 3.827±0.123 \| \| **CD133-** \| 0.365±0.058 \| 1.04±0.093 \| |  |  |
| --- | --- | --- | --- | --- | --- | --- | --- | --- | --- | --- | --- | --- | --- | --- | --- | --- | --- | --- | --- | --- | --- | --- | --- | --- | --- | --- |

Fig. S1


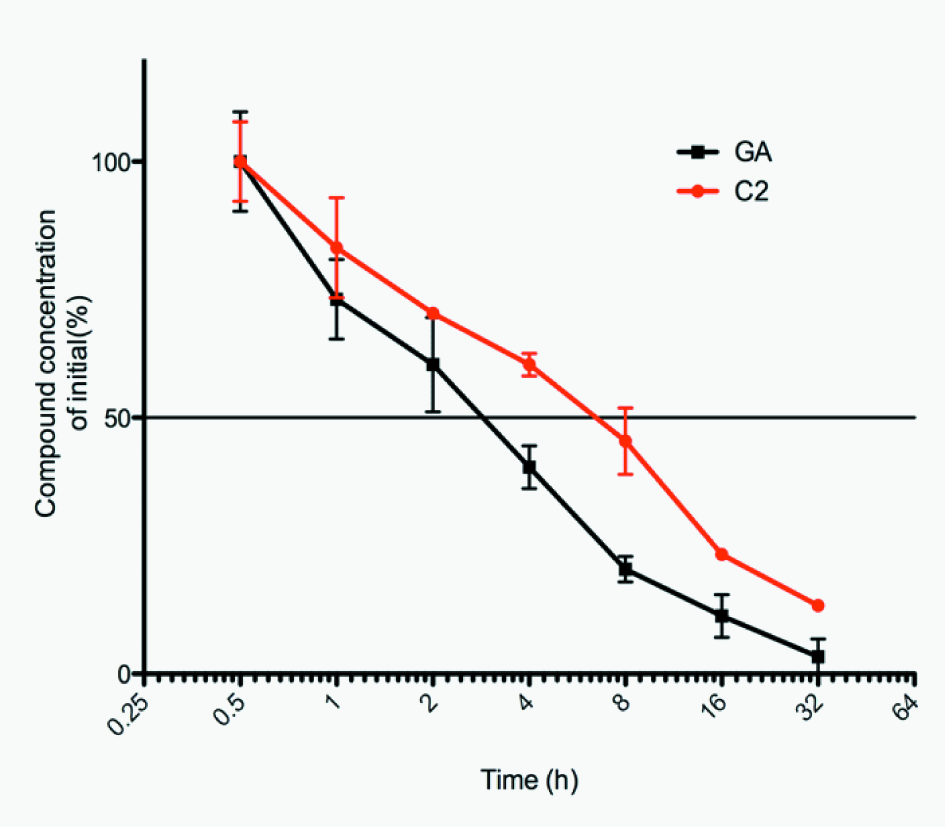


**Fig. S1.** The half-life of C2 and GA was detected by metabolic stability assay combined with LS/MS/MS assay. GA or C2 were incubated with rat liver microsomes for indicated time and the concentration was determined with LC/MS/MS. The half life of GA and C2 was 3.1 and 6.3 h, respectively.

Fig. S2


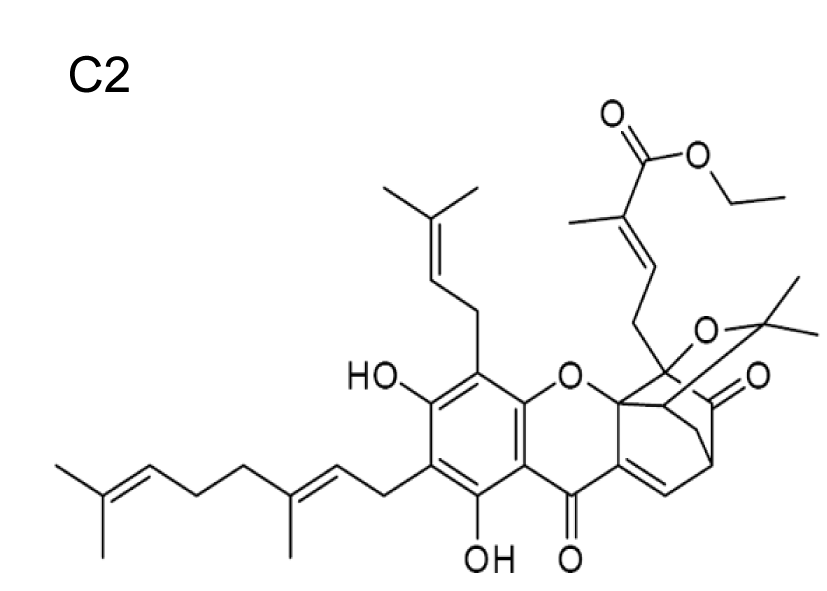


**Fig. S2** Chemical structure of C2.
